# Effects of riociguat on right ventricular size and function in pulmonary arterial hypertension (RIVER II): a prospective, phase IV study

**DOI:** 10.1186/s12931-026-03843-8

**Published:** 2026-07-28

**Authors:** Satenik Harutyunova, Jonathan Heinz, Nicola Benjamin, Lena Brückner, Faruk Sehic, Benjamin Egenlauf, Antonio Cittadini, Alberto M Marra, Ekkehard Grünig, Panagiota Xanthouli

**Affiliations:** 1 Centre for Pulmonary Hypertension, Thoraxklinik-Heidelberg gGmbH at Heidelberg University Hospital and Translational Lung Research Centre Heidelberg (TLRC), Member of the German Centre for Lung Research (DZL), Roentgenstraße 1, Heidelberg, 69126 Germany; 2 Department of Pneumology and Critical Care Medicine, Thoraxklinik-Heidelberg gGmbH at Heidelberg University Hospital, Roentgenstr. 1, Heidelberg, 69126 Germany; 3 Department of Translational Medical Sciences, ’’Federico II“University of Naples, ’’Federico II“ University Hospital and School of Medicine, Naples, Italy; 4 Department of Internal Medicine V: Hematology, Oncology and Rheumatology, Heidelberg University Hospital, INF 410, Heidelberg, 69120 Germany

**Keywords:** Pulmonary arterial hypertension, Riociguat, Right-heart size and function, Echocardiography

## Abstract

**Background:**

Pulmonary arterial hypertension (PAH) is characterized by right ventricular (RV) pressure overload, dilatation and dysfunction, which are key prognostic determinants. While riociguat improves exercise capacity and hemodynamics, prospective data on reverse remodeling remain limited. This prospective, phase IV study evaluated the effects of riociguat on right-heart size, function, hemodynamics, exercise capacity and safety in PAH patients.

**Methods:**

Treatment-naïve or pretreated PAH patients were enrolled. Patients receiving phosphodiesterase-5 inhibitors (PDE5i) could be switched to riociguat upon clinical indication. The primary endpoint was the change in right atrial (RA) and RV area at 24 weeks. Secondary endpoints included additional echocardiographic, clinical, exercise, hemodynamic and laboratory parameters, as well as quality-of-life (QoL) and safety.

**Results:**

Thirty patients (61.2 ± 14.5 years; 76.7% male; 24 PDE5i-pretreated) were enrolled. Marked right-heart dilatation and impaired RV function were prominent at baseline. At week 24, riociguat significantly reduced RA (Δ -3.17 ± 5.91 cm²; *p* = 0.006) and RV area (Δ -6.00 ± 3.80 cm²; *p* < 0.0001). Furthermore, RV-fractional area change, 6-minute walking distance and functional class improved significantly. In patients with invasive follow-up, cardiac index increased significantly, with favorable trends in further parameters. N-terminal pro-brain natriuretic peptide and QoL remained unchanged. The study terminated early following the decision by the supplier Merck/MSD; the termination was not safety related. Riociguat was generally well tolerated with no new safety concerns.

**Conclusion:**

Riociguat therapy resulted in a statistically significant improvement in right ventricular and atrial size and function with further improvements in exercise capacity and functional class. This prospective trial confirms the findings of previous retrospective studies and supports riociguat as an effective treatment option to improve RV geometry and performance.

**Trial registration:**

This trial was registered on clinicaltrials.gov with the ClinicalTrials.gov ID NCT04954742.

**Supplementary Information:**

The online version contains supplementary material available at 10.1186/s12931-026-03843-8.

## Introduction

Pulmonary arterial hypertension (PAH) is characterized by progressive vascular remodeling, increase in pulmonary arterial pressure, pulmonary vascular resistance (PVR) and right ventricular (RV) overload that can ultimately lead to right-heart failure and death [1]. At the time of diagnosis, most patients present with marked enlargement of the right ventricular (RV) and right atrial (RA) area as well as impaired cardiac output (CO) [1].

Riociguat is an established therapeutic option for both PAH and chronic thromboembolic pulmonary hypertension (CTEPH) and is endorsed by current treatment guidelines [1, 2]. In pivotal registration trials, riociguat improved significantly the primary endpoint, 6-minute walking distance (6MWD), as well as several secondary endpoints including PVR, N-terminal pro–brain natriuretic peptide (NT-proBNP) levels, World Health Organization functional class (WHO-FC), time to clinical worsening, and Borg dyspnea score [2, 3]. Despite the clinical relevance of these trials, data on effects of riociguat on right-heart size and function are limited, as the phase III riociguat trial protocols did not include echocardiographic assessments to investigate changes in RV or RA area [2, 3]. Current pulmonary hypertension (PH) treatment guidelines identify RA area as one of the most robust echocardiographic determinants of clinical outcome [1, 4, 5]. Changes in RA and RV area are both strongly correlated with prognosis in PAH [6, 7]. Alterations in RV area are reported to reflect RV function and performance and are associated with right-heart reverse remodeling [8, 9]. These parameters have been shown to be inversely associated with mortality and serve as independent prognostic predictors of survival in PAH [9–11]. Subsequent retrospective analyses have suggested that riociguat may induce reverse remodeling of the right heart, with reductions in RA and RV areas and improvements in RV systolic function, tricuspid annular plane systolic excursion (TAPSE) and RV fractional area change (RV-FAC) observed after 3 to 12 months of therapy in both PAH and CTEPH [12, 13]. Given that right-heart size and function are key determinants of prognosis and treatment response, prospective confirmation of effects of riociguat on cardiac remodeling in patients with advanced PAH and dilated right-heart is clinically warranted.

The aim of this study was thus to prospectively evaluate the effects of 24-week riociguat therapy on right-heart size and function using echocardiography in PAH/CTEPH patients. Further evaluation included assessments of right-heart function, exercise capacity, laboratory parameters, such as NT-proBNP, and the hemodynamic properties assessed by right-heart catheterization (RHC) in patients with PAH.

## Materials and methods

### Study design and procedures

This prospective, open-label, single center, phase IV clinical trial evaluated the effects of riociguat on echocardiographic measures of right-heart size and function, as well as on invasive hemodynamics and exercise capacity in patients with precapillary PH. Adult patients with either PAH or CTEPH with a mean pulmonary artery pressure (mPAP) > 20 mmHg and PVR > 2 Wood Units (WU), pulmonary arterial wedge pressure (PAWP) ≤ 15 mmHg [1], were eligible for inclusion.

Participants were either newly diagnosed and treatment-naïve with respect to PAH-specific therapy or pre-treated according to current guidelines [1]. Pre-treated individuals were required to be on a stable regimen for > 8 weeks prior to enrolment. Transition from PDE5i to riociguat was performed in cases of PDE5i intolerance or clinical indication, with appropriate washout periods (sildenafil: 24 h; tadalafil: 48 h) prior to riociguat initiation. On-site study visits were conducted at baseline, week 12 and 24. Riociguat titration was performed according to the established dose-titration algorithm after telephone consultation. The last follow-up took place no later than week 28.

Echocardiographic assessments were performed at each on-site visit to evaluate right-heart morphology and function, including RA and RV area, RV-FAC, and TAPSE. Hemodynamic measurements via right-heart catheterization (RHC) were obtained within six months prior the screening visit and at week 24 under standardized conditions following guideline recommendations. Echocardiography was performed by experienced sonographers including supervision for cross checks of imaging via PACS to limit bias. Clinical and functional evaluations were conducted at each visit including 6MWD, WHO-FC, laboratory measurements including NT-proBNP levels. Cardiopulmonary exercise testing (CPET) and stress Doppler echocardiography (SDE) were conducted in a subset of participants, mainly limited by technical constraints following the COVID-19 pandemic.

Further details regarding the study design, in- and exclusion-criteria and the statistical analysis are provided in the Supplementary Material: text in black.

### Endpoints and statistical analysis

#### Primary endpoint

The primary efficacy endpoint was the evaluation of change from baseline (i.e. treatment initiation) to week 24 in the RA and RV area. The primary analysis set was the intention-to-treat (ITT) set. The per-protocol analysis (PPA) was supportive (sensitivity analysis). The main comparison was the difference between baseline and week 24. 95% confidence intervals of treatment difference were also calculated. The primary comparison was a paired two-sided student’s t-test. P-values < 0.05 were considered as statistically significant. For the primary endpoint, the threshold for the p-value was 0.025 (according to alpha-splitting for two components).

For the secondary endpoints improvement in WHO functional class, the change in the 6-minute walk distance, in additional echocardiographic parameters, as well as correlation between echocardiographic parameters, in pulmonary function tests, invasively measured hemodynamic parameters, CPET, laboratory parameters such as NT-proBNP, and quality-of-life were tested exploraatively using paired student’s t-tests, McNemar (Bowker) tests in case of frequency data or Wilcoxon signed rank test in case of nonparametric data and reported with 95% confidence intervals, mean and standard deviation of the mean. Parameters were analyzed as change from baseline to visit 3. A nonparametric sensitivity analysis assigning worst case values to a patient who had missing RHC assessment due to worsening of PAH was performed according to a reviewer’s wish. To assess right-heart reverse remodeling (RHRR), echocardiographic criteria were applied in accordance with Badagliacca et al., including RA-area reduction > 1.3 cm^2^, RV-area reduction > 2.45cm^2^ and left-ventricular-eccentricity-index (LV-EI) reduction > 0.12 [9].

#### Safety

Patients were included in the safety and ITT analyses if they received at least one dose of study medication. Laboratory parameters, vital signs and assessment of adverse events (AE) and serious adverse events (SAE) served as safety parameters. Major protocol deviations were defined as failure to meet in-/exclusion criteria and/or non-adherence to the study treatment protocol, including compliance < 80% or > 120% (corresponding to an overdose). Analyses were performed with IBM SPSS V29.0 (IBM, Somers, New York).

## Results

### Subject disposition and baseline characteristics

**Fig. 1:**
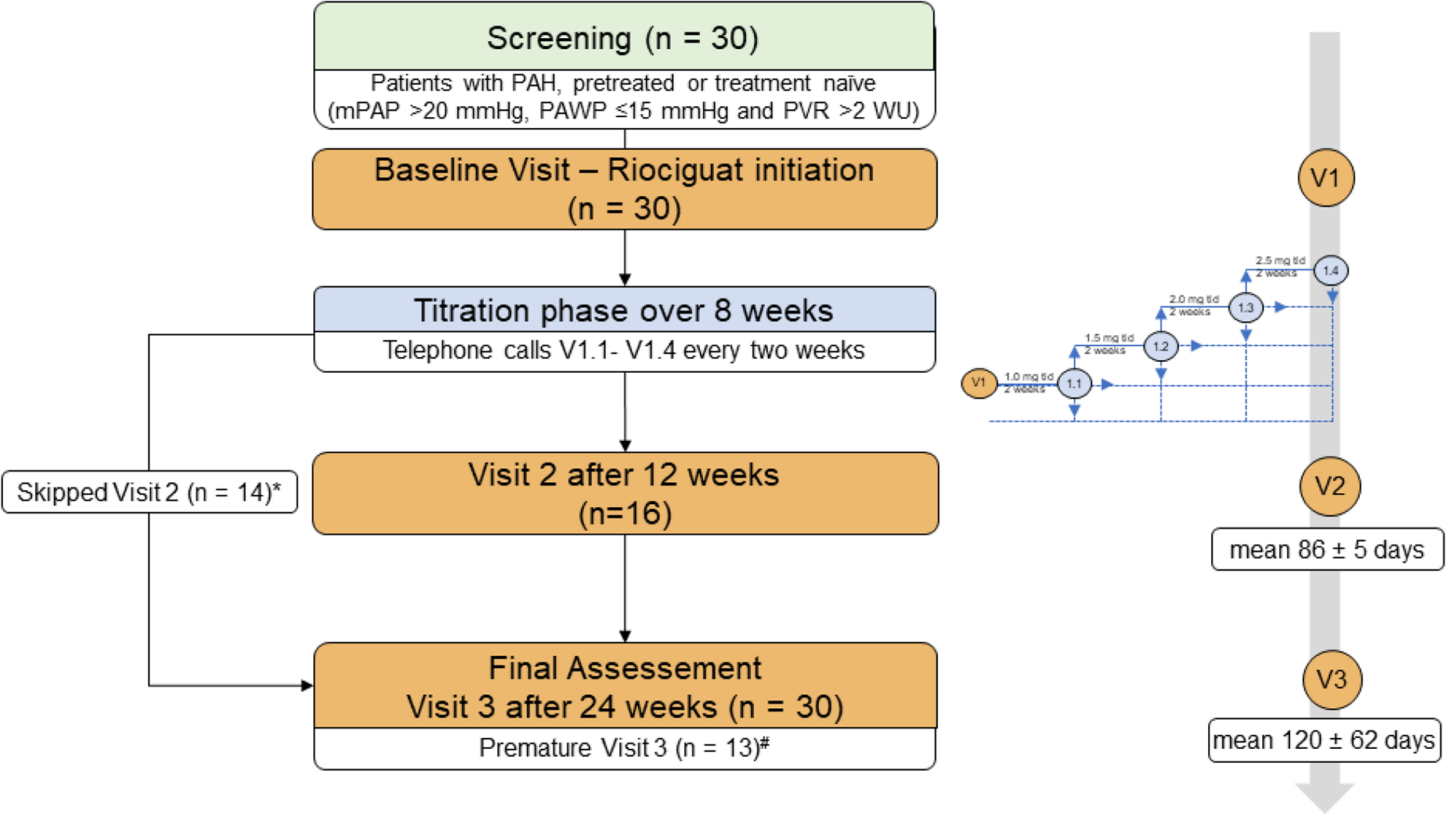
Study flow-chart. Out of 30 patients who were enrolled into the study, 14 skipped the interim visit after 12 weeks (visit 2). All patients performed a final assessment of the study (Visit 3, scheduled 24 weeks after baseline), with 13 premature visits. Most of the premature visits were due to the premature termination of the trial. All patients were valid for the intention-to-treat and safety analysis, while two patients had to be excluded from the per-protocol analysis as they did not fulfil all in-/exclusion criteria. *Due to personal circumstances (n=1), due to early termination of the study (n=9), early termination of trial medication (n=3), hospitalisation at time of scheduled visit 2 (SAE) (n=1). ^#^Early termination of the study (n=9), early termination of trial medication (n=3), patient request (n=1). mPAP: mean pulmonary arterial pressure; PAH: Pulmonary arterial hypertension; PAWP: pulmonary arterial wedge pressure; PVR: Pulmonary vascular resistance; tid: thrice daily; WU: Wood units

Overall, 30 patients were enrolled in the study and received riociguat as mono- or combination therapy over a period of 24 weeks between 13 April 2022 and 30 May 2025 (Fig. 1; Table 1). Most patients had idiopathic PAH (*n* = 23), followed by hereditary PAH (*n* = 4) and drug- or toxin-induced PAH (*n* = 1). Two patients had associated forms of PAH, one case with portal hypertension (*n* = 1) and one associated with connective tissue disease (*n* = 1). No patients with CTEPH were enrolled as none of the screened patients fulfilled the inclusion criteria. The majority of participants were male (76.7%) and had a mean age of 61.23 ± 14.52 years, with a mean PH duration of 3.59 ± 6.98 years (Table 1).

**Table 1:** Demographics

Parameter [Unit]	Baseline					
	*N* = 30					
	Mean ± SD or *n* and %			Median		*n*
Female no. [%]	7.00		23.30			7
Age [years]	61.23	±	14.52		64.00	30
Duration of PAH [years]	3.59	±	6.98		1.60	30
PAH etiology						
Idiopathic	23		76.67	%		
Hereditary	4		13.33	%		
Drug- and toxin-induced PAH	1		3.33	%		
Associated PAH with	2		6.67	%		
Portal hypertension	1		3.33	%		
Connective-tissue disease	1		3.33	%		
Cardiovascular comorbidities						
Arterial hypertension	16		53.33	%		
Coronary heart disease	14		46.66	%		
Obesity	5		16.67	%		
Diabetes mellitus	7		23.33	%		
WHO FC no.						
II	2		6.66	%		
III	25		83.33	%		
IV	3		10.00	%		
Pulmonary hemodynamics						
RAP [mmHg]	6.13	±	3.33		5.00	30
sPAP [mmHg]	68.10	±	19.73		72.00	30
dPAP [mmHg]	25.53	±	6.02		25.00	30
mPAP [mmHg]	40.97	±	9.44		40.50	30
PAWP [mmHg]	9.07	±	3.06		9.00	30
CO [l/min]	5.25	±	1.56		4.80	30
CI [l/min/m^2^]	2.62	±	0.74		2.45	30
PVR [WU]	6.72	±	3.47		5.95	30
SvO_2_ [%]	70.47	±	6.98		71.00	30

*CI* Cardiac index, *CO* Cardiac output, *dPAP* Diastolic pulmonary arterial pressure, *mPAP* mean pulmonary arterial pressure, *PAH* Pulmonary arterial hypertension, *PAWP* Pulmonary arterial wedge pressure, *PVR* Pulmonary vascular resistance, *RAP* mean right atrial pressure, *SD* Standard deviation, *sPAP* Systolic pulmonary arterial pressure, *SvO*_2_ venous oxygen saturation, *WU* Wood Units, *WHO-FC* World Health Organization-functional class

A switch from PDE5i therapy was performed in 22 patients receiving dual therapy with PDE5i and ERA, four patients were previously treated with sildenafil and 18 with tadalafil. In some cases, treatment decisions favouring targeted PAH monotherapy were influenced by the presence of idiopathic PAH with comorbidities. Three patients were newly diagnosed and treatment-naive prior to initiating riociguat. One patient was on monotherapy with tadalafil, which was switched to riociguat. In three patients riociguat was added to a previous monotherapy consisting of ERAs in two patients and an inhaled prostacyclin analogue in one patient. In one patient under triple therapy with PDE5i, ERA and an oral prostacyclin receptor agonist the PDE5i was switched to riociguat.

Echocardiographic assessments demonstrated marked right-heart enlargement and impaired RV function in most patients. At baseline, the mean RA area was 22.50 ± 6.14 cm², and the mean RV area was 26.10 ± 5.50 cm² (Table 1). Qualitative assessment of RV systolic function revealed that 26 of 30 patients had moderate-to-severe impairment at baseline, while three patients showed mild impairment and one patient had normal RV function. Baseline laboratory assessments revealed elevated NT-proBNP levels with a mean concentration of 1555.04 ± 3165.73 ng/l. The mean 6MWD was 359.83 ± 123.01 m and most patients were functionally impaired with 93.3% classified as WHO-FC ≥ III (6.70% FC II, 83.30% FC III, 10.00% FC IV). Hemodynamic assessment (mean 0.71 ± 1.43 months before baseline) revealed severe impairment, with a mean mPAP of 40.97 ± 9.44 mmHg and mean PVR of 6.72 ± 3.47 WU (Table 1).

Patients included in the study showed no evidence of clinically relevant pulmonary disease at diagnosis. As shown in Table 2, pulmonary function parameters including total lung capacity (TLC), forced vital capacity (FVC), and forced expiratory volume in 1 s (FEV1) were largely preserved, whereas diffusing capacity of the lung for carbon monoxide (DLCO) was reduced, likely reflecting severe pulmonary vascular disease and prolonged disease duration.

**Table 2:** Baseline measurements and changes of echocardiographic parameters, hemodynamics, 6MWD and further clinical parameters

Parameter [Unit]	Baseline					Changes at visit 3						
	Mean ± SD			Median	*n*	Mean ± SD			*n*	95% Confidence interval		*p*-value* (t-test)
										Lower	Upper	
Echocardiography at rest												
Estimated sPAP [mmHg]	57.83	±	17.04	57.00	30	-4.33	±	15.32	30	-10.05	1.39	0.132
RA area [cm^2^]	22.50	±	6.14	20.50	30	-3.17	±	5.91	30	-5.37	-0.96	**0.006**
RV area [cm^2^]	26.10	±	5.50	26.00	30	-6.00	±	3.80	30	-7.42	-4.58	**< 0.0001**
TAPSE [cm]	2.15	±	0.42	2.20	30	0.16	±	0.55	30	-0.04	0.37	0.114
LV-EI	1.33	±	0.24	1.35	28	-0.12	±	0.31	28	-0.24	0.00	0.052
FAC [%]	17.23	±	5.14	18.00	22	6.32	±	8.99	22	2.33	10.30	**0.003**
IVC diameter [cm]	1.64	±	0.46	1.80	30	-0.05	±	0.67	30	-0.30	0.20	0.685
IVC collapse [%]	56.29	±	21.92	50.00	24	1.38	±	22.95	24	-8.32	11.07	0.772
Left atrial diameter [cm]	3.30	±	0.52	3.40	28	0.11	±	0.56	28	-0.11	0.33	0.323
PA diameter [cm]	2.96	±	0.75	3.00	24	-0.21	±	0.78	24	-0.54	0.12	0.203
TAPSE/sPAP [mm/mmHg]	0.41	±	0.16	0.37	30	0.08	±	0.21	30	0.00	0.16	**0.044**
Hemodynamics at rest												
RAP [mmHg]	6.18	±	3.66	5.00	17	0.24	±	4.32	17	-1.99	2.46	0.825
sPAP [mmHg]	63.41	±	17.71	63.00	17	3.29	±	14.91	17	-4.37	10.96	0.376
dPAP [mmHg]	25.59	±	5.08	25.00	17	-0.12	±	5.80	17	-3.10	2.86	0.934
mPAP [mmHg]	39.88	±	8.40	38.00	17	0.65	±	7.10	17	-3.00	4.30	0.712
PAWP [mmHg]	9.18	±	2.74	9.00	17	1.76	±	5.65	17	-1.14	4.67	0.216
CO [l/min]	5.45	±	1.72	5.20	17	0.41	±	0.81	17	-0.01	0.83	0.054
CI [l/min/m^2^]	2.69	±	0.86	2.80	17	0.24	±	0.44	17	0.01	0.46	**0.040**
PVR [WU]	6.41	±	3.80	5.00	17	-1.04	±	2.89	17	-2.52	0.45	0.158
SvO_2_ [%]	71.88	±	6.51	73.00	17	0.53	±	4.56	17	-1.81	2.87	0.638
6MWD												
6MWD [m]	369.00	±	119.96	394.50	28	41.00	±	57.94	28	18.53	63.47	**0.001**
SpO_2_ [%] test end	87.29	±	8.81	89.00	28	-0.25	±	5.10	28	-2.23	1.73	0.797
Blood gas analysis												
SaO_2_ [%]	93.90	±	2.52	94.00	24	-2.31	±	4.76	24	-4.32	-0.30	**0.026**
PaO_2_ [mmHg]	69.00	±	10.71	68.00	29	-3.28	±	9.45	29	-6.87	0.32	0.072
PaCO_2_ [mmHg]	33.99	±	4.75	33.40	29	-0.90	±	3.69	29	-2.31	0.50	0.197
pH	7.43	±	0.04	7.43	29	0.01	±	0.03	29	0.00	0.02	0.091
Short-Form-36 Health Survey												
Physical functioning	54.64	±	27.35	57.50	28	3.21	±	14.48	28	-2.40	8.83	0.250
Physical role function	50.89	±	45.38	62.50	28	4.46	±	36.68	28	-9.76	18.69	0.525
Pain	85.32	±	19.96	100.00	28	-5.57	±	21.38	28	-13.86	2.72	0.179
General health perception	51.50	±	19.68	48.50	28	0.93	±	18.95	28	-6.42	8.28	0.797
Vitality	52.50	±	18.18	52.50	28	0.71	±	12.74	28	-4.23	5.66	0.769
Social functioning	76.96	±	23.73	88.00	28	5.79	±	26.27	28	-4.40	15.97	0.254
Emotional role function	78.57	±	35.42	100.00	28	-9.54	±	43.40	28	-26.36	7.29	0.255
Mental well-being	70.71	±	19.57	76.00	28	4.14	±	18.39	28	-2.99	11.28	0.244
Physical summation score	58.79	±	19.72	58.00	28	0.79	±	12.91	28	-4.22	5.79	0.750
Mental summation score	65.96	±	17.96	66.50	28	0.50	±	17.27	28	-6.20	7.20	0.879
Pulmonary function tests												
FVC [%predicted]	84.54	±	11.37	81.00	30	0.91	±	14.90	30	-4.65	6.47	0.740
FEV_1_ [%predicted]	81.95	±	11.85	81.85	30	-1.26	±	12.21	30	-5.82	3.30	0.576
TLC [%predicted]	91.04	±	12.54	89.50	30	2.12	±	16.55	30	-4.06	8.30	0.488
Residual volume [%predicted]	99.29	±	26.40	92.05	30	1.34	±	23.68	30	-7.50	10.18	0.759
DLCO [%predicted]	52.55	±	21.34	56.50	28	0.22	±	7.13	28	-2.54	2.99	0.871
DLCO/VA [%predicted]	60.86	±	23.20	64.85	28	1.19	±	6.51	28	-1.33	3.71	0.342
Laboratory												
Hemoglobin [g/dl]	15.48	±	2.27	15.50	27	-0.54	±	1.18	27	-1.01	-0.07	**0.026**
Hematocrit	0.46	±	0.06	0.44	27	-0.02	±	0.04	27	-0.03	0.00	**0.015**
Creatinine [mg/dl]	1.05	±	0.24	1.02	28	0.03	±	0.12	28	-0.02	0.08	0.200
Urea [mg/dl]	38.14	±	18.40	30.50	28	-1.50	±	10.03	28	-5.39	2.39	0.436
SGOT [U/l]	27.43	±	22.46	24.50	28	-5.46	±	19.05	28	-12.85	1.92	0.141
SGPT [U/l]	29.50	±	33.63	19.50	28	-6.46	±	28.55	28	-17.54	4.61	0.241
Bilirubin [mg/dl]	0.82	±	0.51	0.70	28	0.17	±	0.35	28	0.03	0.30	**0.019**
NTproBNP [ng/l]	1555.04	±	3165.73	434.00	28	-21.04	±	2144.27	28	-852.50	810.43	0.959

*CI* Cardiac index. *CO* Cardiac output. *CRP* C-reactive protein, *DLCO* Diffusing capacity of the lung for carbon monoxide, *DLCO/VA* Diffusing capacity of the lung for carbon monoxide divided by the alveolar volume, *dPAP* diastolic pulmonary arterial pressure, *FAC* RV fractional area change, *FVC* Forced vital capacity, *FEV*_1_ Forced expiratory volume in 1 s, *IVC* Inferior vena cava diameter; *LV-EI* Left ventricular eccentricity index, *mPAP* mean pulmonary arterial pressure, *NT-proBNP* N-terminal pro-brain natriuretic peptide, *PA* Pulmonary artery, *PaCO*_2_Partial pressure of carbon dioxide, *PaO*_2_ Partial pressure of oxygen, *PAWP* pulmonary arterial wedge pressure, *PVR* Pulmonary vascular resistance, *RA* Right atrium, *RAP* mean right atrial pressure, *RV* Right ventricle, *SaO*_2_ oxygen saturation, *SD* Standard deviation, *sPAP* systolic pulmonary arterial pressure, *SGOT* Serum glutamic oxaloacetic transaminase, *SGPT* Serum glutamic pyruvic transaminase.*SpO*_2_ partial oxygen saturation, *SvO*_2_:venous oxygen saturation, *TAPSE* Tricuspid annular plane systolic excursion, *TLC* Total lung capacity, *WU* Wood Units, *6MWD* Six-minute walking distance

*p-values were derived from student’s t-tests

statistically significant values (p<0.05) are highlighted in bold

Two patients failed to meet all inclusion criteria (signs of parenchymal lung disease *n* = 1; not stable on PH-specific treatment and unspecific treatment *n* = 1) and were therefore excluded from the per-protocol analysis (*n* = 28). Treatment compliance was within the predefined target range for all participants.

The study terminated early following the decision by the supplier Merck/MSD; the termination was not related to safety issues or study conduct. Overall, thirteen patients terminated the study prematurely, including nine who completed a premature final assessment due to supplier-driven early study termination (Fig. 1). Instead of performing visit 2, these nine patients were included in the final assessment (visit 3) earlier (Fig. 1). One patient requested early study termination, and three discontinued study medication prematurely due to adverse events. Overall, fourteen participants skipped visit 2 (Fig. 1).

### Primary endpoint right-heart size

**Fig. 2:**
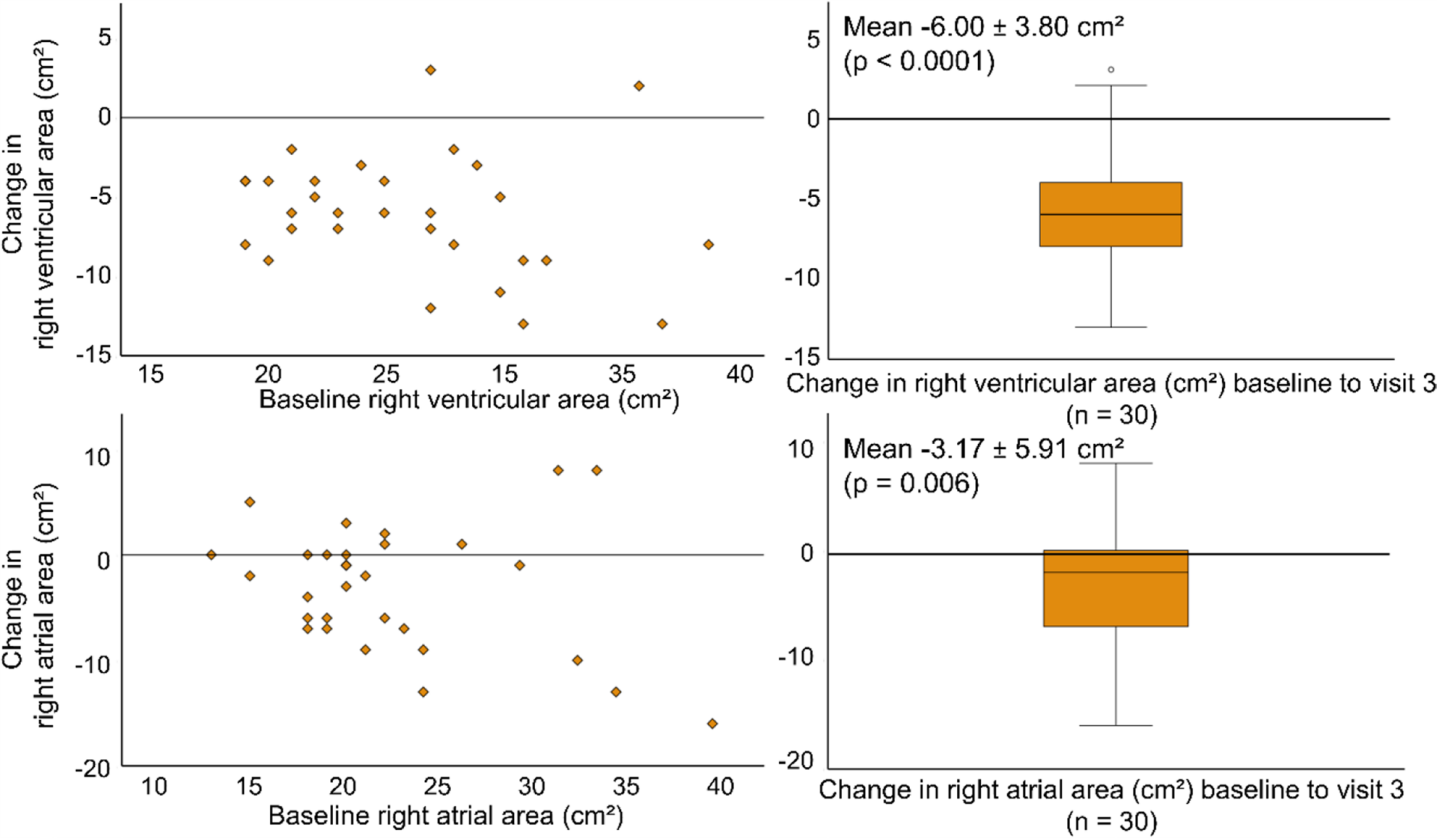
Change in primary endpoint right atrial and right ventricular area. Scatterplots at the left side are presenting baseline values und respective individual changes for each patient. Changes during the study seemed to be independent from baseline. The boxplots show the observed mean changes in RV and RA area at visit 3 compared to baseline. The bottom and top of the boxes indicate the first and third quartiles and the lines inside the boxes are the median values. The whiskers that extend from each box indicate the range of values outside of the interquartile range, but are within a distance less than or equal to 1.5 times interquartile range. Outliers are defined as values with a distance of more than 1.5 times interquartile range and are indicated by circles. RA (*p* = 0.006) and RV area (*p* < 0.0001) significantly improved throughout the study. A p-value < 0.025 was considered significant for the study, as the primary endpoint consisted of both components (alpha splitting). Reduction of right-heart size was independent from baseline values

Treatment with riociguat resulted in a statistically significant and clinically meaningful reduction in both RA and RV area from baseline to visit 3. The mean reduction in RA area was 3.17 ± 5.91 cm^2^ (*p* = 0.006, Table 2. Fig. 2). Similarly, RV area decreased significantly, with a mean change of -6.00 ± 3.80 cm^2^ (*p* < 0.0001) from baseline, corresponding to a reduction of 13.4% and 23.4%, respectively (Table 2; Fig. 2). Per protocol analysis showed robust results for both components of the primary endpoint (RA area *p* = 0.004, RV area *p* < 0.0001). Results were confirmed in a sensitivity analysis only including patients who switched riociguat from a PDE5i with significant reduction of RA (*p* = 0.010) and RV area (*p* < 0.001).

### Secondary endpoints

#### Echocardiographic changes

**Fig. 3:**
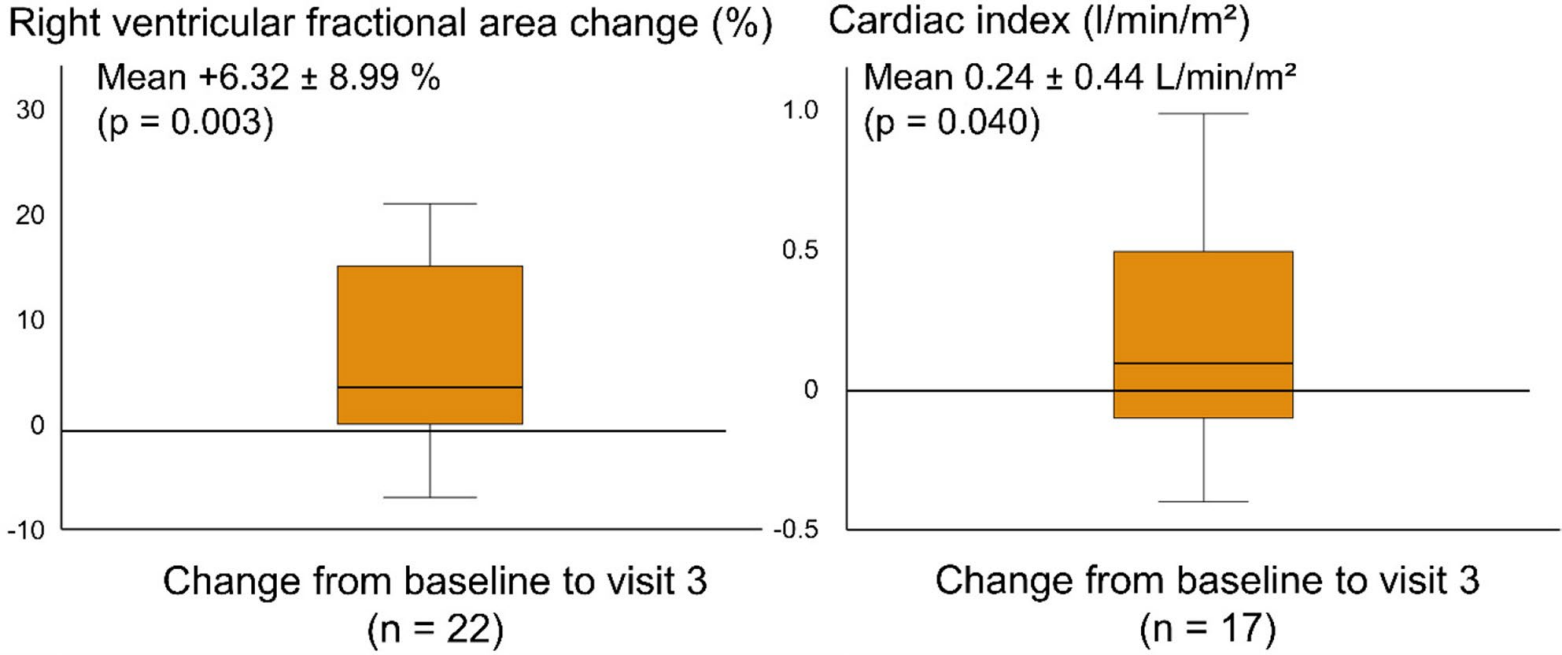
Secondary endpoints: changes in right-heart function. The boxplots show the observed mean changes in RV-FAC and CI at visit 3 compared to baseline. The bottom and top of the boxes indicate the first and third quartiles and the lines inside the boxes are the median values. The whiskers that extend from each box indicate the range of values outside of the interquartile range, but are within a distance less than or equal to 1.5 times interquartile range. Outliers are defined as values with a distance of more than 1.5 times interquartile range and are indicated by circles. Riociguat significantly improved RV-FAC (*p* = 0.003) and invasively measured CI at rest (*p* = 0.040) at week 24

Echocardiographic assessment demonstrated a significant improvement in RV systolic function under riociguat. RV-FAC increased by a mean of 6.32 ± 8.99% (*p* = 0.003) (Table 2; Fig. 3). Qualitative evaluation of RV function showed improvements in eight patients, stability in 21, and deterioration in one patient. Although this trend suggested functional improvement, it did not reach statistical significance *(p* = 0.072, McNemar test). TAPSE/sPAP showed a significant improvement of 0.08 ± 0.21 mm/mmHg (*p* = 0.044). Further echocardiographic parameters, including TAPSE, sPAP and inferior vena cava (IVC) diameter, did not significantly change compared to baseline (Table 2). Additionally, parameters of RHRR were assessed: Overall, 89.3% of participants (*n* = 25) fulfilled at least one criterion of reverse remodeling under treatment, while 28.6% (*n* = 8) fulfilled all three criteria of RHRR (Table 3). When only including patients who switched riociguat from a PDE5i, sPAP (*p* = 0.038) significantly reduced and RV-FAC (*p* = 0.003) showed a significant increase from baseline until the end of the study. LV-EI reduced in trend (*p* = 0.052), but was significantly reduced at the interim visit (*p* = 0.004). Other parameters did not significantly differ between baseline and follow-up in this subcohort.

**Table 3:** Number of patients fulfilling criteria of right-heart reverse remodeling

	Number of patients		
	*n* = 28		**%**
Criterion of RHRR			
RA-area reduction > 1.3 cm^2^	16	53.3	**%**
RV-area reduction > 2.45 cm^2^	26	86.7	%
LV-EI reduction > 0.12	14	50	%
Number of RHRR criteria fulfilled			
0	3	10.7	**%**
1	6	21.4	%
2	11	39.3	%
3	8	28.6	**%**

*LV-EI* Left ventricular eccentricity index, *RA* Right atrium, *RHRR* Right-heart reverse remodeling, *RV* Right ventricle

### Invasive hemodynamic changes, WHO-FC, 6MWD, CPET, quality-of-life

Invasive hemodynamic measurements obtained by RHC during 24 weeks follow-up were available for 17 patients (Table 2). There was a statistically significant increase in CI with a mean change of 0.24 ± 0.44 l/min/m² (*p* = 0.040) (Fig. 3). CO and PVR showed numerical but statistically non-significant changes, with CO increasing by 0.41 ± 0.81 l/min (*p* = 0.054) and PVR decreasing by 1.04 ± 2.89 WU (*p* = 0.158). No other invasively measured hemodynamic parameters demonstrated significant changes compared with baseline (Table 2). One patient did not perform RHC assessment at visit 3 due to worsening of PAH. A non-parametric sensitivity analysis (Wilcoxon signed rank test) did not show any significant changes in invasive hemodynamics when this patient was assigned worst case values for the respective parameters.

**Fig. 4:**
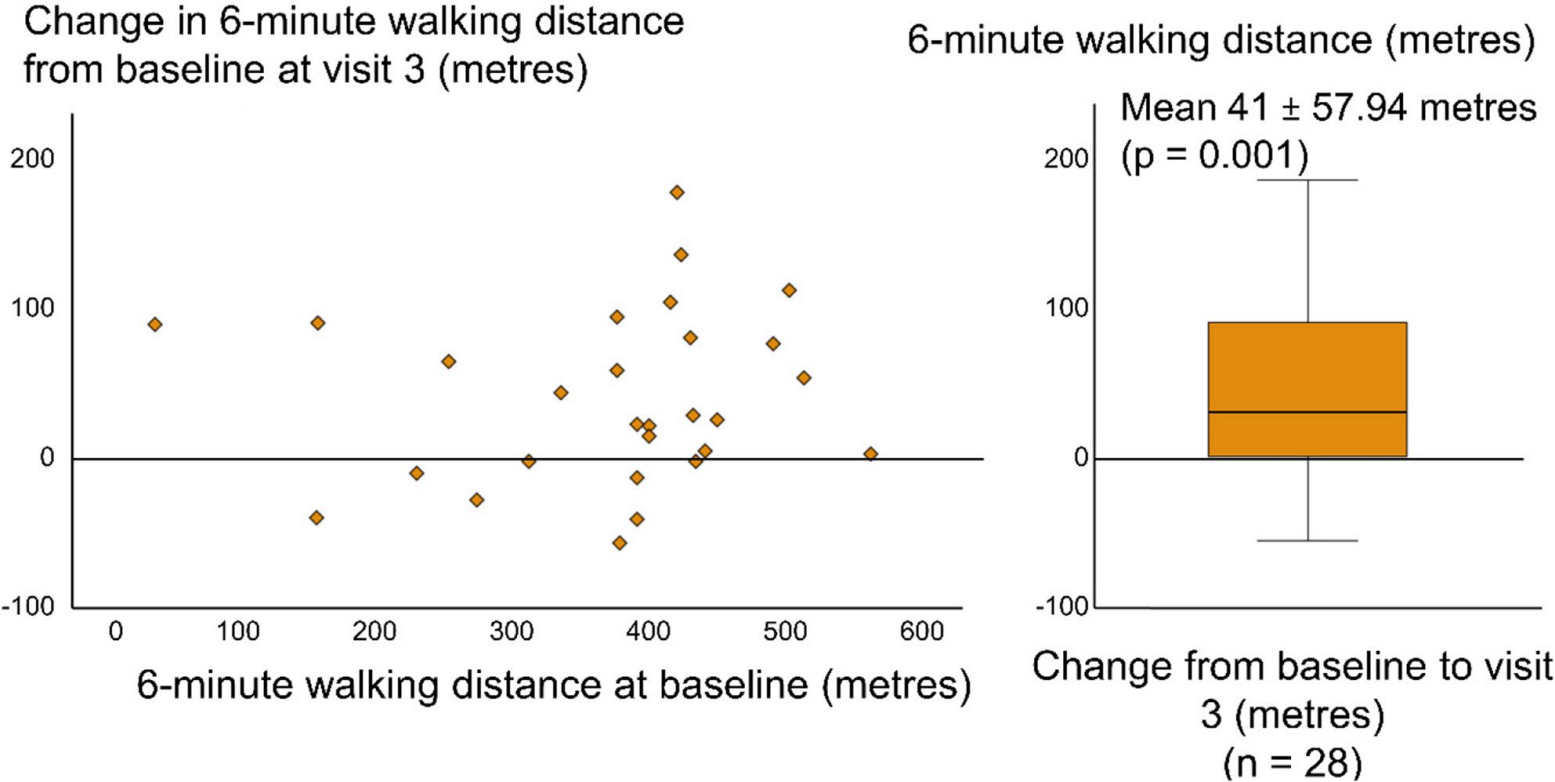
Secondary endpoints: changes in 6-minute walking distance. The scatterplot at the left side is presenting baseline values und respective individual changes of 6MWD for each patient. Changes during the study seemed to be independent from baseline. The boxplot shows the observed mean change 6-MWD at visit 3 compared to baseline. The bottom and top of the boxes indicate the first and third quartiles and the lines inside the boxes are the median values. The whiskers that extend from each box indicate the range of values outside of the interquartile range, but are within a distance less than or equal to 1.5 times interquartile range. Outliers are defined as values with a distance of more than 1.5 times interquartile range and are indicated by circles. 6-MWD showed a significant and clinically relevant improvement during riociguat treatment (*p* = 0.001)

**Fig. 5:**
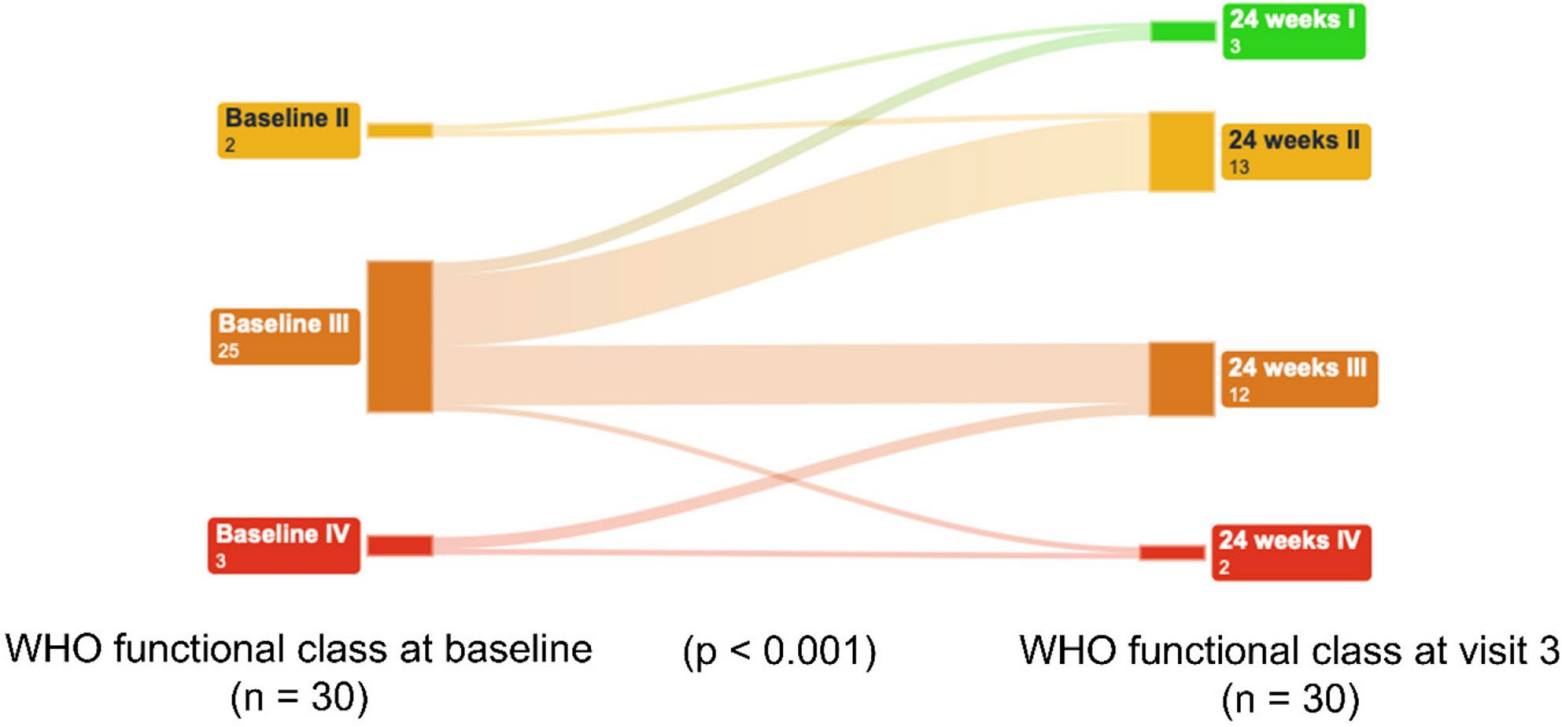
Secondary endpoints: changes in WHO functional class. The Sankey diagram shows the proportion of patients with improved, stable or worsened WHO-FC from baseline to visit 3. WHO-FC improved in seventeen patients (56.67%), remained stable in twelve (40%) and deteriorated in one patient (3.33%, *p* < 0.001)

At visit 3, treatment with riociguat resulted in a statistically significant and clinically meaningful improvement in WHO-FC (Wilcoxon signed rank test, *p* < 0.001) compared with baseline. WHO-FC improved in 17 patients (56.7%), remained stable in 12 (40.0%) and deteriorated in one patient (3.3%) (Wilcoxon signed rank test, *p* < 0.001, Fig. 5). Exercise capacity also improved significantly, with a mean increase in 6MWD of 41.00 ± 57.94 m (*p* = 0.001) (Table 2; Fig. 4). Patient-reported quality-of-life outcomes assessed by the SF-36 did not change significantly (Table 2). Including only patients who switched from a PDE5i, results showed an increase in CI (*p* = 0.040) and 6MWD (*p* < 0.001).

### Laboratory tests and pulmonary functional parameters

At visit 3, there were no statistically significant changes in NT-proBNP levels, pulmonary function parameters, or diffusion capacity for carbon monoxide under riociguat treatment (Table 2). Among laboratory parameters, a small but statistically significant increase in total bilirubin was observed, with a mean change of 0.17 ± 0.35 mg/dl (*p* = 0.019). All other liver function parameters remained stable, without significant changes during treatment. In addition, hemoglobin levels decreased modestly (Δ -0.54 ± 1.18 g/dL, *p* = 0.026) accompanied by a reduction in hematocrit of approximately 2 ± 4% (*p* = 0.015). No relevant bleeding events occurred during the trial. The reduction of hemoglobin and hematocrit (significant but within normal range) did not fulfill the criteria of anemia and was thus not listed as AE and not associated with the medication. Oxygen saturation at the end of the trial was also significantly reduced by 2.31 ± 4.76% (*p* = 0.026); however, partial pressure of oxygen (PaO₂) remained within an acceptable physiological range and did not indicate clinically relevant hypoxemia, also partial oxygen saturation (SpO_2_) after 6MWD showed no significant changes. These changes were deemed not clinically relevant and not related to study medication by the treating physician. Patients who switched from a PDE5i reduced in FEV1 (*p* = 0.033), SpO_2_ (*p* = 0.026), hemoglobin (*p* = 0.026), hematocrit (*p* = 0.015) bilirubin (*p* = 0.019) from baseline until the end of the study.

### Safety and tolerability

An overview of AE occurring in at least 5% of patients is provided in Table 4. The overall incidence of AE during the study was 96.67%, with 29 participants experiencing at least one AE. Most events were of mild or moderate intensity and resolved spontaneously or with minimal medical intervention. AE included dizziness (23.3%), hypotension (16.7%), edema (16.7%), heartburn (43.3%), and palpitations (16.7%). Respiratory infections occurred in 20% of participants. These events are consistent with the established safety profile of riociguat in patients with PAH. No new or unexpected safety signals were observed throughout the study period.

**Table 4:** Adverse events ≥ 5% and Serious adverse events (SAE)

	*AE > 5% / SAE*			proportion related to riociguat		
	*n* = 30		%	*n* = 30		%
Adverse events ≥ 5%						
Cardiac disorders						
Palpitation	5	16.67	%	4	13.33	%
Tachycardia	2	6.67	%	1	3.33	%
Hypotension	5	16.67	%	4	13.33	%
Dizziness	7	23.33	%	6	20.00	%
Edema	5	16.67	%	0	0.00	%
Unspecified circulatory system disorder	2	6.67	%	2	6.67	%
Respiratory, thoracic and mediastinal disorders						
Cough	3	10.00	%	0	0.00	%
Dyspnea	8	26.67	%	5	16.67	%
Respiratory insufficiency	2	6.67	%	1	3.33	%
Gastrointestinal disorders						
Constipation	2	6.67	%	1	3.33	%
Heartburn	13	43.33	%	12	40.00	%
General disorders and administration site conditions						
Fatigue	4	13.33	%	1	3.33	%
Infections and infestations						
Covid-19 infection	2	6.67	%	0	0.00	%
Respiratory Infection	6	20.00	%	0	0.00	%
Metabolism and nutrition disorders						
Iron deficiency	2	6.67	%	0	0.00	%
Vitamin B12 deficiency	2	6.67	%	0	0.00	%
Vitamin D deficiency	2	6.67	%	0	0.00	%
Musculoskeletal and connective tissue disorders						
Muscle cramps	2	6.67	%	1	3.33	%
Pain	4	13.33	%	1	3.33	%
Nervous system disorders						
Headache	7	23.33	%	5	16.67	%
Psychiatric disorders						
Sleep disorders	2	6.67	%	0	0.00	%
Vascular disorders						
Epistaxis	2	6.67	%	1	3.33	%
Serious adverse events*						
Vitreous hemorrhage	1	3.33	%	0	0.00	%
Corneal edema	1	3.33	%	0	0.00	%
Fluid overload	1	3.33	%	0	0.00	%
PAH progression	1	3.33	%	0	0.00	%
Respiratory infection	1	3.33	%	0	0.00	%
Infection with hypoxemia	1	3.33	%	0	0.00	%

*All meeting the criterion of hospitalization or prolongation of hospitalization, all SAE not related with study medication

Two patients discontinued treatment prematurely due to AE (gastrointestinal symptoms and respiratory insufficiency in one participant; headache and hypotension in the other). These events were deemed mild to moderate, associated with the study medication and resolved after discontinuation. Three patients discontinued the study due to lack of tolerability. 17 patients were on riociguat until week 24 while 9 patients were on riociguat until the trial was stopped by the sponsor due to non-safety related reasons. Thus, 87% tolerated the medication until the end of the trial.

A total of six SAE were reported in three patients, all meeting the criteria for (prolonged) hospitalization (Table 4). One patient experienced severe deterioration of PAH during the titration phase, accompanied by respiratory insufficiency and infection, leading to hospitalization and study drug discontinuation. Another participant was hospitalized twice for ophthalmologic complications and later withdrew from the study on his own request and due to the initial SAE. A third participant was hospitalized due to fluid overload and treated with intravenous diuretics. None of the SAE were considered related to the study medication. Overall, riociguat was well tolerated, and no new or unexpected safety signals were identified. There were no deaths during the study.

## Discussion

RIVER II is the first clinical trial to prospectively evaluate the effects of riociguat treatment on right-heart structure and function, as well as clinical outcomes in PAH patients over 24 weeks. The initiation of 24-week treatment with riociguat significantly improved the primary endpoint RA and RV area by 13.4% and 23.4%, respectively. Improvements were also observed in WHO-FC, 6MWD, CI, and RV-FAC. At baseline, patients presented with advanced disease, characterized by marked enlargement of RA and RV areas and predominantly moderate-to-severe RV systolic dysfunction. These findings demonstrate a favorable effect of riociguat on right-heart size in this population.

### Structural and functional changes of the right heart

Right-heart function and size are key prognostic factors in PAH [1]. The observed reduction of right-heart size under riociguat treatment may suggest a potential to delay or prevent a progression to right-heart failure by improving RV contractility. These findings reinforce the role of riociguat not only as a vasodilator, but as a therapy possibly capable of restoring right-heart geometry and performance in patients with persistent RV dilation despite prior treatment or PDE5i therapy. Clinically, these improvements were accompanied by better exercise capacity and symptom relief, translating into meaningful patient benefit.

Previous studies have demonstrated that changes in right-heart chamber size are associated with reverse remodelling and improved RV systolic performance [9, 10].

The RIVER II study extends this evidence by demonstrating direct structural RA and RV improvements under riociguat treatment in a prospective setting. The magnitude of remodeling observed in RIVER II, particularly the pronounced reduction in RV area compared to earlier reports [13, 14], may reflect the more severely dilated baseline phenotype and the prospective, standardized follow-up design of this study.

An improvement in RV performance was observed in this study, as indicated by the significant change in RV-FAC. RV-FAC reflected more accurately the impaired RV pump function than TAPSE. While TAPSE assesses only longitudinal RV motion, FAC reflects global RV contraction and may therefore better represent RV pump function in PAH [15]. Moreover, a significant improvement of TAPSE/sPAP as a parameter of RV-PA coupling could be shown in patients under riociguat, indicating improvement of contractile RV-performance [16].

The high predictive value of echocardiographically assessed RHRR could be demonstrated compared to other functional and hemodynamic parameters in a large previous study including PAH patients [9]. In this study, 89.3% of patients met at least one criterion of RHRR under therapy, suggesting favourable effects of riociguat on RHRR.

The predominance of male patients in this cohort, despite PAH typically being more prevalent in women, likely reflects the inclusion of patients with dilated right-heart chambers requiring treatment escalation, for whom RA dilatation was one of the criteria supporting a switch to riociguat, as male PAH patients have been shown to exhibit larger right-heart dimensions [17].

The present findings are consistent with results from previous pivotal trials [2, 3, 18], which established the efficacy of riociguat in improving exercise capacity, WHO-FC, and hemodynamics in PAH.

Post-hoc analyses of pivotal studies showed that riociguat increased stroke volume index, RV power, and cardiac efficiency while reducing pulmonary arterial elastance, confirming a hemodynamic pattern of improved RV afterload and energetics [19]. Patients who achieved a favorable stroke-volume-to-RA pressure phenotype experienced better long-term outcomes in the extension trials from phase III riociguat studies [19]. Experimental models have shown reversal of RV hypertrophy and fibrosis [20–22], while clinical studies, demonstrated increases in TAPSE, RV-FAC, and cardiac index following riociguat initiation or switch from PDE5i [13, 23]. The results of the current study corroborate previous retrospective analyses reporting reductions in RA and RV size and improvements in RV systolic function under riociguat therapy [12, 13].

### Changes in hemodynamic, functional capacity, and biomarkers

Hemodynamic improvements of right-heart function in this cohort were accompanied by better clinical and functional outcomes under treatment. These results confirm already existing data [2]. 6MWD increased by > 40 m (*p* = 0.001) (Fig. 4), and WHO-FC improved significantly (p *<* 0.001, Fig. 5) which correlates with previous studies [2]. NT-proBNP levels did not change significantly, possibly reflecting the baseline heterogeneity, the limited sample size and the relatively short follow-up period [23]. Subgroup analyses based on CPET indicated significant improvements in VO₂ and exercise performance (Supplementary Material). Overall, the concordant improvements across echocardiographic, hemodynamic, and functional domains suggest that riociguat effectively supports both pulmonary vascular and RV recovery.

### Safety

Riociguat was safe and generally well tolerated. AE occurred in 29 out of 30 patients, were typically mild to moderate, and resolved spontaneously, consistent with the known safety profile of riociguat. Six SAE occurred in three patients (10%), none of which were considered related to the study medication. Minor laboratory changes (increased bilirubin, small decreases in hemoglobin and hematocrit) were statistically significant but not clinically relevant. No new or unexpected safety signals emerged, and the overall safety profile was consistent with previous reports [2, 3].

### Limitations

This was a single center, open-label study with a limited sample size and no control group, which restricts direct comparison to placebo or standard therapy. Therefore, the findings should be interpreted as demonstrating an association between riociguat therapy and improvements in right-heart size and function rather than establishing a causal relationship. Furthermore, the absence of a comparator arm precludes direct comparison with alternative treatment strategies or with the natural course of disease. Early termination for non-safety-related reasons by the supplier further reduced study treatment exposure, potentially limiting effect size due to shortened study duration in several patients. The lack of blinding may have introduced assessment bias, particularly in subjective echocardiographic and functional endpoints. Nonetheless, the consistent improvements across objective imaging, hemodynamic, and functional domains provide supportive internal validity for the observed effects. Due to a reduced sample size for RHC assessments, the interpretability of the invasive hemodynamic assessment is limited. Though PH due to left heart or lung disease were ruled out by exclusion criteria, data on smoking history would have been desirable for the interpretation of results.

## Conclusion

In this prospective, open-label, phase IV trial, treatment with riociguat significantly improved the primary endpoint right-heart size measured by echocardiographic RA and RV area and secondary endpoints including RV function, exercise capacity and WHO-FC, indicating meaningful clinical effects. Riociguat had a favorable benefit–risk ratio. These findings confirm and extend the results of previous studies and suggest riociguat as a safe and effective therapeutic option capable of improving right ventricular geometry and performance in patients with PAH and right-heart enlargement. Further studies are warranted to confirm these observations and to elucidate the mechanisms and long-term prognostic implications of riociguat-induced right-heart reverse remodeling.

## Supplementary Information

Supplementary Material 1.

## Data Availability

Data is available upon reasonable request to the corresponding author.

## Abbreviations

6MWD: 6-minute walking distance
AE: Adverse event
CI: Cardiac index
CO: Cardiac output
CPET: Cardio pulmonary exercise testing
CTEPH: Chronic thromboembolic pulmonary hypertension
DLCO: Diffusing capacity of the lung for carbon monoxide
FC: Functional class
FEV_1_: Forced expiratory volume in 1s
FVC: Forced vital capacity
ITT: Intention to treat
IVC: Inferior vena cava
LV-EI: Left ventricular eccentricity index
mmHg: Millimetres of mercury
mPAP: Mean pulmonary arterial pressure
NT-proBNP: N-terminal prohormone of brain natriuretic peptide
PAH: Pulmonary arterial hypertension
PAWP: Pulmonary arterial wedge pressure
PDE5i: Phosphodiesterase-5 inhibitors
PH: Pulmonary hypertension
PVR: Pulmonary vascular resistance
QoL: Quality of life
RA: Right atrium
RAP: Right atrial pressure
RHC: Right heart catheterization
RHRR: Right-heart reverse remodeling
RV: Right ventricle
RV-FAC: Right ventricular fractional area change
SAE: Serious adverse event
SD: Standard deviation
SDE: Stress Doppler echocardiography
SF-36: 36-item short form survey as part of the medical outcome study
sGC: Soluble guanylate cyclase
sPAP: Systolic pulmonary arterial pressure
TAPSE: Tricuspid annular plane systolic excursion
TLC: Total lung capacity
TRV: Tricuspid regurgitation velocity
VO2: Volume of oxygen uptake
WHO: World Health Organization
WU: Wood units
