# Supplementary material for: Effects of riociguat on right ventricular size and function in pulmonary arterial hypertension (RIVER II): a prospective, phase IV study
Source: Respir Res. 2026 Jul 28;27:296. doi: 10.1186/s12931-026-03843-8 (PMC13425866; doi:10.1186/s12931-026-03843-8)
Supplement: Supplementary file 1 — Supplementary Material 1. [file 12931_2026_3843_MOESM1_ESM.docx]

**Effects of Riociguat on RIght VEntriculaR size and function in pulmonary arterial hypertension (RIVER II): a prospective, phase IV study**

Satenik Harutyunova MD^1,2^, Jonathan Heinz^1,2^, Nicola Benjamin Dr. sc. hum.^1,2^, Lena Brückner PhD^1,2^, Faruk Sehic MPharm^1,2^, Benjamin Egenlauf MD^1,2^, Antonio Cittadini MD^3^, Alberto M Marra MD^3^, Ekkehard Grünig MD^1,2^, Panagiota Xanthouli MD^1,2,4*^

^1^Centre for Pulmonary Hypertension, Thoraxklinik-Heidelberg gGmbH at Heidelberg University Hospital and Translational Lung Research Centre Heidelberg (TLRC), Member of the German Centre for Lung Research (DZL), Heidelberg, Germany
^2^Department of Pneumology and Critical Care Medicine, Thoraxklinik-Heidelberg gGmbH at Heidelberg University Hospital, Heidelberg, Germany

^3^Department of Translational Medical Sciences, "Federico II“ University of Naples, "Federico II“ University Hospital and School of Medicine, Naples, Italy

^4^Department of Internal Medicine V: Hematology, Oncology and Rheumatology, Heidelberg University Hospital, Heidelberg, Germany

***Corresponding author:**

Assoc. Prof. Panagiota Xanthouli, MD

Center for Pulmonary Hypertension

Thoraxklinik Heidelberg gGmbH at Heidelberg University Hospital

Roentgenstraße 1, 69126 Heidelberg, Germany and

Department of Internal Medicine V: Hematology, Oncology and Rheumatology

University Hospital Heidelberg

INF 410, Heidelberg, Germany

Tel: +49 6221 396 8270

ORCID IDs: 0000-0002-7743-2472 (PX)

E-Mail: [panagiota.xanthouli@med.uni-heidelberg.de](mailto:panagiota.xanthouli@med.uni-heidelberg.de)

**Table of contents**

[**Methods** 3](#_Toc222213742)

[**Inclusion Criteria** 3](#_Toc222213743)

[**Exclusion Criteria** 6](#_Toc222213744)

[**Criteria for Removal or Withdrawal** 8](#_Toc222213745)

[**Ethical approval** 10](#_Toc222213746)

[**Statistical analysis** 10](#_Toc222213747)

[**S5: Study schedule** 12](#_Toc222213748)

[**Results of cardiopulmonary exercise testing** 14](#_Toc222213749)

[**S6: Baseline values and changes of cardiopulmonary exercise testing parameters at visit 3** 16](#_Toc222213750)

[**Graphical abstract** 17](#_Toc222213751)

## **Methods**

### **Inclusion Criteria**

| 1. ≥18 years of age at time of inclusion. 2. Male and female patients with: 3. symptomatic PAH with a mean pulmonary artery pressure (mPAP) >20 mmHg and pulmonary vascular resistance (PVR) ≥2 Wood Units (WU), pulmonary arterial wedge pressure (PAWP) ≤15 mmHg (Group I / Nice Clinical Classification of Pulmonary Hypertension) or 4. CTEPH (Group IV / Nice Clinical Classification of Pulmonary Hypertension) defined as one of the following options:  - inoperable measured at least 3 months after start of full anticoagulation and mPAP >20 mmHg and PVR ≥2 WU, PAWP ≤15 mmHg or - persisting or recurrent PH after pulmonary endarterectomy (mPAP >20 mmHg and PVR ≥2 WU, PAWP ≤15 mmHg measured at least 6 months after surgery (acc. to Simonneau et al. 2018).  1. Patients who are either 2. **treatment** **naïve** (with respect to PAH specific medication) or **pre-treated with an endothelin receptor antagonist** and/or a **prostacyclin** and/or a **prostacyclin analogue** (according to upfront combination treatment)^[[1]](#footnote-1)^ or 3. **pre-treated with phosphodiesterase type 5 inhibitor (PDE-5)** with or without combination treatment with an endothelin receptor antagonist and/or prostacyclin analogue. Pre-treated patients need to be stable on PDE5i for at least two months prior to Visit 1. “Stable” is defined as no change in the type of PDE5i and the respective daily dose. PDE5i has to be stopped (sildenafil 24h, tadalafil 48h) before switching to riociguat. The switch from PDE5i to riociguat is due to clinical indication, particularly when the patient´s risk-profile remained in intermediate risk group despite adequate initial treatment including PDE5i (defined as at least 3 of the following parameters: clinical signs of progression, persistent WHO-FC III, 6MWD between 165-440m, peak V02 11-15ml/min/kg (35-65% predicted), NTproBNP 300-1400 ng/l, RA-area 18-26cm2,RAP 8-14mmHg, CI 2,0-2,4 l/min) or in case of PDE5i intolerance. Any decision to switch will be made by the clinicians at a regular clinical follow-up visit. 4. Unspecific treatments which may also be used for the treatment of PH such as oral anticoagulants, diuretics, digitalis, calcium channel blockers or oxygen supplementation are permitted. However, treatment with anticoagulants (if indicated) must have been started at least 1 month before **visit 1** in patients with PAH. 5. RHC results must not be older than 6 months at screening (will be considered as baseline values) and must have been measured in the participating centre under standardized conditions (refer to the study specific Swan Ganz catheterization manual). If the respective measurements have not been performed in context with the patient’s regular diagnostic workup, they have to be performed as a part of the study during the pre-study phase (after the patient signed the informed consent). 6. Women without childbearing potential defined as postmenopausal women aged 50 years or older, women with bilateral tubal ligation, women with bilateral ovariectomy, and women with hysterectomy can be included in the study. 7. Women of childbearing potential can only be included in the study if all of the following applies (listed below): 8. Negative serum pregnancy test at Screening and a negative urine pregnancy test at study start (visit 1). 9. Agreement to undertake monthly urine pregnancy tests during the study and up to at least 30 days after study treatment discontinuation. These tests should be performed by the patient at home. 10. Agreement to follow the contraception scheme below from Screening until at least 30 days after study treatment discontinuation.  \| Option 1 \| or \| Option 2  One Method from this list \| \| or \| \| Option 3 \| or \| \| Option 4 \| \| --- \| --- \| --- \| --- \| --- \| --- \| --- \| --- \| --- \| --- \| \| Tubal sterilization  (occlusion or  ligation of tubes  at least 6 weeks  prior to  Screening) \| \|  \| Oral*,  Implantable*,  Transdermal*, or  Injectable*  hormonal  contraceptives  Intrauterine  Devices \|  \| Sterilization of the  male partner with  documented post-vasectomy  confirmation of the  absence of sperm  in the ejaculate  **PLUS one**  **method from this**  **list**  Oral*,  Implantable*,  Transdermal*, or  Injectable*  hormonal  contraceptives  Intrauterine  devices  Diaphragm,  female condom  cervical cap,  partner’s use of a  condom \| \|  \| True abstinence  from intercourse  with a male  partner only when  this is in line with  the preferred  lifestyle of the  subject. \| \|   * If a hormonal contraceptive is chosen from this group, it must have been taken for at least 28 days prior to study start (visit 1).   1. Patients who are able to understand and follow instructions and who are able to participate in the study for the entire period. 2. Patients must have given their written informed consent to participate in the study after having received adequate previous information and prior to any study-specific procedures. |
| --- | --- | --- | --- | --- | --- | --- | --- | --- | --- | --- | --- | --- | --- | --- | --- | --- | --- | --- | --- | --- |

### **Exclusion Criteria**

1. Pregnant women, or breast-feeding women, or women of childbearing potential not able or willing to comply with study-mandated contraception methods specified above.
2. Patients with PH specific treatment who are not stable for at least 2 months before screening.
3. Patients with a medical disorder, condition, or history of such that would impair the patient's ability to participate or complete this study in the opinion of the investigator.
4. Patients with underlying medical disorders with an anticipated life expectancy below 2 years (e.g. active cancer disease with localized and/or metastasized tumour mass).
5. Patients with a history of severe or multiple drug allergies
6. Patients with hypersensitivity to the investigational drug or any of the excipients.
7. Patients unable to perform a valid 6MWD test (e.g. orthopaedic disease, peripheral artery occlusive disease, which affects the patient´s ability to walk).
8. The following specific medications for concomitant treatment of PH or medications which may exert a pharmacodynamic interaction with the study drug are not allowed:
9. Specific phosphodiesterase inhibitors (e.g. sildenafil or tadalafil): may be switched to riociguat but not be given in addition to the study drug
10. or unspecific phosphodiesterase inhibitors (e.g. dipyridamole, theophylline)
11. NO donors (e.g. nitrates)
12. Pulmonary diseases exclusions
13. Moderate to severe bronchial asthma or COPD (Forced Expiratory Volume <60% predicted) or severe restrictive lung disease (Total Lung Capacity < 70% predicted) and/or defined as if high resolution computed tomography shows >20% parenchymal lung disease.
14. Severe congenital abnormalities of the lungs, thorax, and diaphragm.
15. Clinical or radiological evidence of Pulmonary-Veno-Occlusive Disease (PVOD) or Pulmonary Capillary Haemangiomatosis (PCH) or PH and idiopathic interstitial pneumonia (PH-IIP)
16. Cardiovascular exclusions:
17. Uncontrolled arterial hypertension (systolic blood pressure >180 mmHg and /or diastolic blood pressure >110 mmHg).
18. Systolic blood pressure <95 mmHg (permitted and monitored closely if due to the study drug side effects).
19. Left heart failure with an ejection fraction less than 40%.
20. Hypertrophic obstructive cardiomyopathy.
21. Severe proven or suspected coronary artery disease according to investigators opinion (patients with Canadian Cardiovascular Society Angina Classification class 2-4, and/or requiring nitrates, and/or myocardial infarction within the last 3 months before Visit 1).
22. Clinical evidence of symptomatic atherosclerotic disease (e.g. peripheral artery disease with reduced walking distance, history of stroke with persistent neurological deficit etc).

Exclusions related to disorders in organ function:

1. Clinically relevant hepatic dysfunction indicated by:

- bilirubin >2 times upper limit normal
- and / or hepatic transaminases >3 times upper limit normal
- and / or signs of severe hepatic insufficiency (e.g. impaired albumin synthesis with an albumin < 32 g/l, hepatic encephalopathy > grade 1a: *West Haven Criteria of Altered Mental Status In Hepatic Encephalopathy*)

1. Renal insufficiency (glomerular filtration rate <30 ml/min/m^2^ e.g. calculated based on the Cockcroft formula).

### **Criteria for Removal or Withdrawal**

Withdrawal of Subjects

A subject will be withdrawn from the trial treatment for the following reasons:

1. at their own request or at request of their legally acceptable representative
2. if, in the investigator's opinion, continuation of the trial would be detrimental to the subject's well-being
3. occurrence of a severe serious adverse event (SAE) caused by the IMP
4. The participant has a medical condition or personal circumstance which, in the opinion of the investigator, placed the participant at unnecessary risk from continued administration of study treatment.
5. The participant has a confirmed positive serum pregnancy test.

The Coordinating Investigator decides about withdrawal of subjects from trial treatment in case of occurrence of criteria mentioned above. Any decision to continue with the study treatment despite occurrence of any of the withdrawal criteria has to be justified in written form in the case report form (CRF) and in the subject’s medical records.

Patients with a serious adverse event occurring during the study treatment will be followed by the study team until the serious adverse event will have resolved to the pre-study level and/or will have been addressed according to best clinical practice. The patient, either willingly withdrawn from the study or due to premature termination, will be asked thoroughly to complete all examinations scheduled for the final trial day, and these will be performed as far as possible and documented.

In all cases, the reason for withdrawal must be recorded in the CRF and in the subject’s medical records.

In case of withdrawal of a subject at his/ her own request, the reason should be asked for as extensively as possible and documented.

All efforts will be made to follow up the subject.

A subject may/ will be withdrawn from all trial related procedures (including follow-up visits) for the following reasons:

- at their own request or at request of their legally acceptable representative
- non-adherence to the trial-related requirements, which may (have) influence(d) the validity of the trial data

Replacement of Subjects

In total, 30 subjects will be enrolled and included into the intention to treat (ITT) and safety analysis (SA). Subjects who terminate the study prematurely will not be replaced as a 20% drop-out rate is included in this sample size calculation.

## **Ethical approval**

The study protocol and its amendments were approved by the local Ethics Committee of the Medical Faculty of Heidelberg University (Internal number AFmo-139/2021) and the Federal Institute for Pharmaceuticals and Medical Products (BfArM) (EudraCT No. 2020-005462-34; EU CT No. 2023-509696-17-00), as well as registered at [www.clinicaltrials.gov](http://www.clinicaltrials.gov) (NCT04954742) on 29 June 2021. The study was in line with the Declaration of Helsinki. Written informed consent was provided by all participants upfront participation in the trial.

## **Statistical analysis**

**Primary endpoint:** For sample size calculations for both components, data from the retrospective RIVER I study was considered (Marra et al., 2018) and a 99.3% power would be achieved to reject the null hypothesis of RV area (based on a mean effect of 3.0 ± 3.2 cm²) and 85.1% for RA area (based on a mean effect of 2.43 ± 3.8 cm²) according to the paired sample two-tailed Student’s t-test, with a type I error of 0.025 (two-sided) and a valid sample size of 24 patients. For the primary endpoint, a PPA and multiple imputation method with 25 imputations was planned as sensitivity analysis.

All variables were analyzed descriptively with appropriate statistical methods: data (demographic and other baseline characteristics, continuous data at each visit and their change to baseline) are listed and trial summary tables are provided. Descriptive statistics include the usual location and scale statistics (mean, median, standard deviation (SD), standard error, first and third quartiles, minimum and maximum) and 95% confidence limits of mean and median. Frequency tables for qualitative data are provided.

Demographic variables and baseline characteristics were summarized for the ITT population. Further outcome parameters including hemodynamics, symptoms, exercise capacity, lung function and quality of life were tested exploratory and reported with 95% confidence intervals, mean and standard deviation of the mean. Parameters were analyzed as change from baseline to 24 weeks treatment.

## **S5: Study schedule**

The study comprised of a screening phase, a titration phase for 8 weeks following the baseline visit and a maintenance phase until the final assessment after 24 weeks. Patients were regularly contacted by phone during the titration phase. Study visits included assessments of clinical parameters, safety and tolerability.


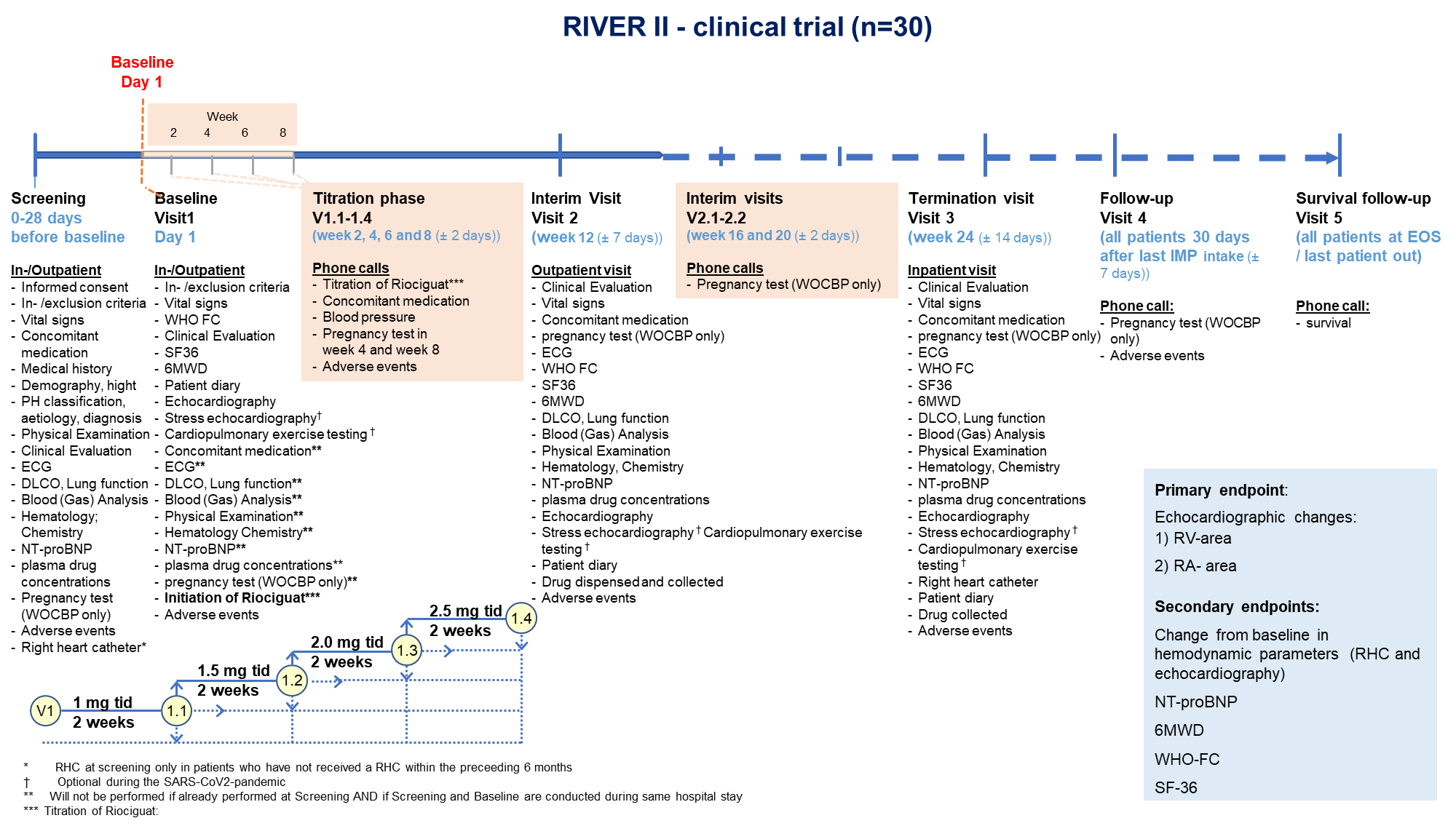


## **Results of cardiopulmonary exercise testing**

Among the 30 patients included in the RIVER II study, a cardiopulmonary exercise test (CPET) was performed in 10 patients (35%) both at baseline and at visit 3, on a semi-reclined cycle ergometer with a stepwise increase of workload by 25 watts every 2 minutes until symptom limitation or meeting a termination criterion (Table S6). Only two patients underwent CPET at baseline and visit 2, an analysis was thus not performed.

There was an overall improvement in exercise capacity following riociguat treatment. Participants achieved a **higher workload** both at the anaerobic threshold (p=0.016) and at maximal exercise (p=0.003), approximately **+30.56 watts at the anaerobic threshold**and**+20.00 watts at peak exercise**compared to baseline, reflecting a clinically meaningful improvement in overall exercise performance and endurance capacity (Table S6). Participants demonstrated a **significant increase in oxygen (O₂) uptake per kilogram (VO₂/kg)** at rest (p=0.021), at the anaerobic threshold (p=0.038), and at peak exercise (p=0.021), indicating an enhanced **O₂** uptake capacity and improved cardiopulmonary efficiency. Similarly, carbon dioxide production (**VCO₂)** at rest (p=0.028), at the anaerobic threshold (p=0.035), and at peak exercise (p=0.021) showed a significant improvement, reflecting improved ventilatory and metabolic response during exertion (Table S6). **Minute ventilation**(VE) at the anaerobic threshold (p=0.021) as well as at peak exercise (p<0.001) was also increased, suggesting improved ventilatory efficiency and higher exercise tolerance under treatment. There was a **significant improvement in O₂ pulse** at rest (p=0.045) and an improvement in trend (p=0.054) at the anaerobic threshold, indicating enhanced stroke volume and **O₂** delivery during exercise (Table S6). There was no significant change of systolic pulmonary arterial pressure at peak workload measured via echocardiography during exercise.

Our findings suggest positive effects of riociguat treatment on exercise capacity, especially on workload**, VO₂/kg, VCO₂, VE and O₂ pulse** in PAH.

## **S6: Baseline values and changes of cardiopulmonary exercise testing parameters at visit 3**

| **Parameter [Unit]** | | **Baseline** | | | | | |  | **Changes at Visit 3** | | | | | | | | |
| --- | --- | --- | --- | --- | --- | --- | --- | --- | --- | --- | --- | --- | --- | --- | --- | --- | --- |
|  |  |  |  |  |  |  |  |  |  |  |  |  |  |  |  |  |  |
|  |  | Mean ± SD | | |  | Median | n |  | Mean ± SD | | | n |  | 95% Confidence Interval | | | p-value* (t-test) |
|  |  |  |  |  |  |  |  |  |  |  |  |  |  | Lower | Upper |  |  |
| **Cardiopulmonary exercise testing** | |  |  |  |  |  |  |  |  |  |  |  |  |  |  |  |  |
|  | VO_2_ at rest [ml/min] | 367.80 | ± | 136.41 |  | 339.00 | 10 |  | -78.00 | ± | 99.39 | 10 |  | -149.10 | -6.90 |  | 0.035 |
|  | VO_2_ at anaerobic (ventilatory) threshold [ml/min] | 937.44 | ± | 429.52 |  | 737.00 | 9 |  | 293.78 | ± | 348.05 | 9 |  | 26.24 | 561.31 |  | 0.035 |
|  | VO_2_ at peak workload [ml/min] | 1248.67 | ± | 456.42 |  | 1304.00 | 9 |  | 171.22 | ± | 158.83 | 9 |  | 49.13 | 293.31 |  | 0.012 |
|  | VO_2_/kg at rest [ml/min/kg] | 4.45 | ± | 1.11 |  | 4.29 | 10 |  | -0.90 | ± | 1.02 | 10 |  | -1.63 | -0.17 |  | 0.021 |
|  | VO_2_/kg at anaerobic (ventilatory) threshold [ml/min/kg] | 10.93 | ± | 4.55 |  | 10.10 | 9 |  | 3.51 | ± | 4.26 | 9 |  | 0.24 | 6.79 |  | 0.038 |
|  | VO_2_/kg at peak workload [ml/min/kg] | 15.06 | ± | 4.24 |  | 15.21 | 9 |  | 2.15 | ± | 2.24 | 9 |  | 0.42 | 3.87 |  | 0.021 |
|  | VCO_2_ at rest [ml/min] | 342.22 | ± | 144.41 |  | 308.00 | 9 |  | -97.22 | ± | 109.29 | 9 |  | -181.23 | -13.22 |  | 0.028 |
|  | VCO_2_ at anaerobic (ventilatory) threshold [ml/min] | 753.13 | ± | 297.37 |  | 735.00 | 8 |  | 445.50 | ± | 484.22 | 8 |  | 40.68 | 850.32 |  | 0.035 |
|  | VCO_2_ at peak workload [ml/min] | 1351.50 | ± | 509.73 |  | 1383.50 | 8 |  | 204.13 | ± | 195.86 | 8 |  | 40.38 | 367.87 |  | 0.021 |
|  | Minute Ventilation (VE) at rest [l/min] | 15.70 | ± | 6.96 |  | 13.00 | 10 |  | 1.80 | ± | 21.36 | 10 |  | -13.48 | 17.08 |  | 0.796 |
|  | Minute Ventilation (VE) at anaerobic (ventilatory) threshold [l/min] | 37.33 | ± | 16.77 |  | 35.00 | 9 |  | 14.67 | ± | 15.42 | 9 |  | 2.81 | 26.52 |  | 0.021 |
|  | Minute Ventilation (VE) at peak workload [l/min] | 57.56 | ± | 16.51 |  | 60.00 | 9 |  | 16.00 | ± | 7.73 | 9 |  | 10.06 | 21.94 |  | <0.001 |
|  | O_2_-pulse at rest [ml] | 4.91 | ± | 1.92 |  | 4.65 | 10 |  | -0.77 | ± | 1.05 | 10 |  | -1.52 | -0.02 |  | 0.045 |
|  | O_2_-pulse at anaerobic (ventilatory) threshold [ml] | 8.71 | ± | 3.48 |  | 7.84 | 9 |  | 1.98 | ± | 2.63 | 9 |  | -0.05 | 4.00 |  | 0.054 |
|  | O_2_-pulse at peak workload [ml] | 9.82 | ± | 3.64 |  | 10.11 | 9 |  | 0.45 | ± | 1.53 | 9 |  | -0.73 | 1.62 |  | 0.408 |
|  | Workload at anaerobic (ventilatory) threshold [Watt] | 69.44 | ± | 37.03 |  | 50.00 | 9 |  | 30.56 | ± | 30.05 | 9 |  | 7.46 | 53.65 |  | 0.016 |
|  | Peak workload [Watt] | 100.00 | ± | 48.59 |  | 112.50 | 10 |  | 20.00 | ± | 15.81 | 10 |  | 8.69 | 31.31 |  | 0.003 |
| **Stress Doppler echocardiography** | |  |  |  |  |  |  |  |  |  |  |  |  |  |  |  |  |
|  | peak systolic pulmonary arterial pressure [mmHg] | 104.70 | ± | 30.62 |  | 97.50 | 10 |  | 12.40 | ± | 38.76 | 10 |  | -15.33 | 40.13 |  | 0.338 |
| *p-values were derived from student's t-tests. An asterisk is given in case of nonparametric testing.  CO_2_: Carbondioxid; O_2_: Oxygen; SD: Standard deviation; sPAP: Systolic pulmonary arterial pressure; VO_2_: Oxygen uptake; VCO_2_: Carbon dioxide production; VE: Minute ventilation. | | | | | | | | | | | | | | | | | |

## **Graphical abstract**


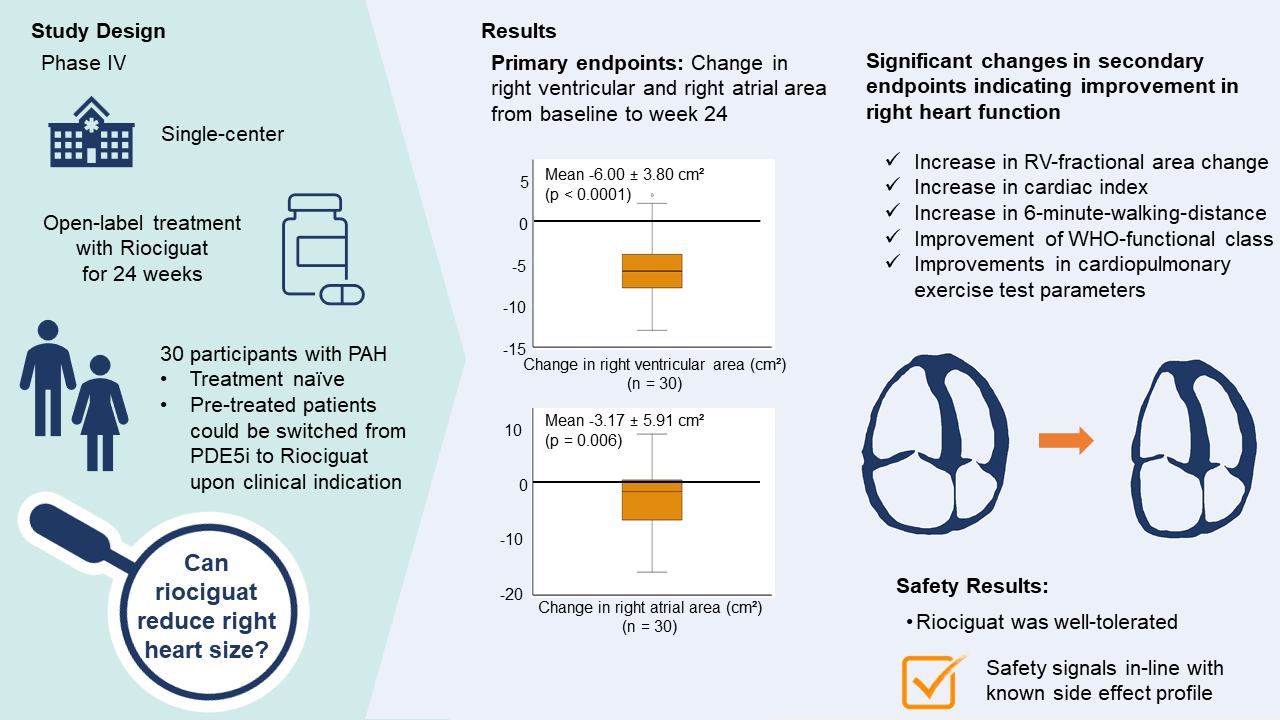


1. Pre-treated patients need to be stable on endothelin receptor antagonists or prostacyclin treatment for at least two months prior to Visit 1. “Stable” is defined as no change in the type of endothelin receptor antagonists or prostacyclin analogue and the respective daily dose. [↑](#footnote-ref-1)
